# Comparative study of cisplatin-based definitive concurrent chemoradiotherapy with S-1 versus paclitaxel for unresectable locally advanced esophageal squamous cell carcinoma

**DOI:** 10.18632/oncotarget.16180

**Published:** 2017-03-14

**Authors:** Min Fang, Tao Song, Xiaodong Liang, Shiliang Lv, Jianbo Li, Hong’en Xu, Limin Luo, Yongshi Jia

**Affiliations:** ^1^ Department of Radiation Oncology, Zhejiang Provincial People's Hospital, Hangzhou 310000, Zhejiang, P. R. China; ^2^ Department of Radiation Oncology, Ningbo Mingzhou Hospital, Ningbo 315000, Zhejiang, P. R. China

**Keywords:** definitive concourrent chemoradiotherapy, esophageal squamous cell carcinoma, S-1, cisplatin, paclitaxel

## Abstract

This study compared the efficiency and safety of definitive concurrent chemoradiotherapy (CCRT) using Paclitaxel plus Cisplatin (TP) versus S-1 plus Cisplatin (CS) in unresectable locally advanced esophageal squamous cell carcinoma (LAESCC). Between January 2009 and December 2013, 203 LAESCC patients were retrospectively reviewed. We performed a propensity score matching analysis; 41 patients treated with the CS regimen were matched 1:1 to patients who received the TP regimen. Patient- and disease-related characteristics were well-balanced between the two groups. The CS group showed significantly better treatment compliance (90.2% vs. 70.7%, *P* = 0.026) and less hospital stay (48 days vs 49 days, *P* = 0.025) over the TP group during the CCRT course. The complete response rate was comparable between the two groups (51.2% vs. 48.8%, *P* = 0.825). The 1- and 3-year overall survival (OS) rates in the TP group were 63.4% and 32.4% compared to 62.8% and 32.1% in the CS group, respectively (*P* = 0.796). The 1- and 3-year progression-free survival (PFS) rates in the TP group were 51.2% and 24.9%, compared to 53.6% and 18.9% in the CS group, respectively (*P* = 0.630). The incidence of severe and total neutropenia in the TP group was significantly higher compared to the CS group (*P* = 0.011 and 0.046, respectively). Multivariate analysis revealed that T stage and the complete response rate were strong prognostic factors associated with OS and PFS. In conclusion, both treatment regimens yielded satisfactory survival outcomes, but the CS regimen could significantly improve treatment compliance, reduce hematological toxicities and lengths of hospital stay. Future prospective studies in large cohorts are highly warranted to confirm the findings in our report.

## INTRODUCTION

Patients presenting with carcinoma of the esophagus continue to represent one of the most significant health problems, and its associated morbidity is still increasing worldwide. Despite advances in diagnosis for early-stage cancers, the majority of patients are diagnosed at advanced stages [1–3]. Definitive concurrent chemoradiotherapy (CCRT) is the standard treatment option for unresectable locally advanced diseases. The seminal phase III trial of the Radiation Therapy Oncology Group (RTOG) 85–01 compared the efficacy of CCRT, which consisted of 5-fluorouracil (5-Fu) plus Cisplatin (CDDP) with radiotherapy (RT) alone and resulted in a long-term survival rate of 26% in the CCRT group. However, the efficacy of this regimen was only approximately 25%–35% while severe and fatal side effects were observed in 44% and 20% of the patients [4]. Attempts have been made to further improve the therapeutic ratio and to reduce toxic reactions for esophageal cancer. Paclitaxel (PTX), a broad-spectrum cytotoxic drug, has been shown to be a promising treatment agent against esophageal cancer and a series of phase II studies has demonstrated favorable response rates and comparable survival outcomes to 5-Fu/CDDP/RT regimens [5, 6]. RTOG 0113 trial, which compared the efficacy of CCRT with the TP (PTX and CDDP) regimen, showed that median survival time was 14.9 months, and the 1- and 2-year OS rates were 69% and 37%, respectively. Severe (grade ≥ 3) side effects were observed in greater than 40% patients [7]. Tang HR et al. reported the median OS and PFS time was 28.5 and 14.7 months, and the 1- and 2-year survival rates were 75% and 54%, respectively with grades 3 and 4 neutropenia occurred in 30.3% and 31.6% in inoperable ESCC patients who received CCRT with a 3-week schedule of TP regimen [8]. Thus, the current National Comprehensive Cancer Network (NCCN) guidelines for esophageal cancer recommend definitive CCRT based on 5-Fu or Taxane for patients who refused surgery or were medically unfit for esophagectomy. However, adverse effects associated with PTX and Cremophor/ethanol, including acute hypersensitivity and neurotoxicity, were still observed in nearly 40% patients, which limited its implementation in the clinic [9].

The compound drug, S-1 (TS-1, Taiho Pharmaceutical), consists of a combination of tegafur-gimeracil-oteracil. S-1 has been widely used in a variety of solid tumors, including esophageal cancer. Preclinical studies indicated that S-1 demonstrates far superior anti-tumor activities than 5-Fu and enhances the sensitivity of cancer cells to the effects of RT [10, 11]. Accumulating clinical evidence also supports the opinion that the combination of CDDP and S-1(CS) represents a viable treatment regimen for esophageal cancer [12, 13]. In studies performed in Japan, the CS regimen combined with RT in the setting of CCRT achieved encouraging response rates of 64.4%-–89.7%. In addition, toxicities associated with CS are modest, which enabled their concomitant use with RT for esophageal cancer patients [14, 15].

Considering the high-grade evidence for the use of the CS regimen combined with RT in locally advanced esophageal squamous cell carcinoma (LAESCC) is in shortage, we performed this retrospective study to compare the clinical outcomes of CS versus TP with RT for unresectable LAESCC, focusing on treatment compliance, response rate, toxicity, and survival outcomes through propensity score matching (PSM) analysis.

## RESULTS

### Demographic variables of the enrolled patients

**Figure 1:**
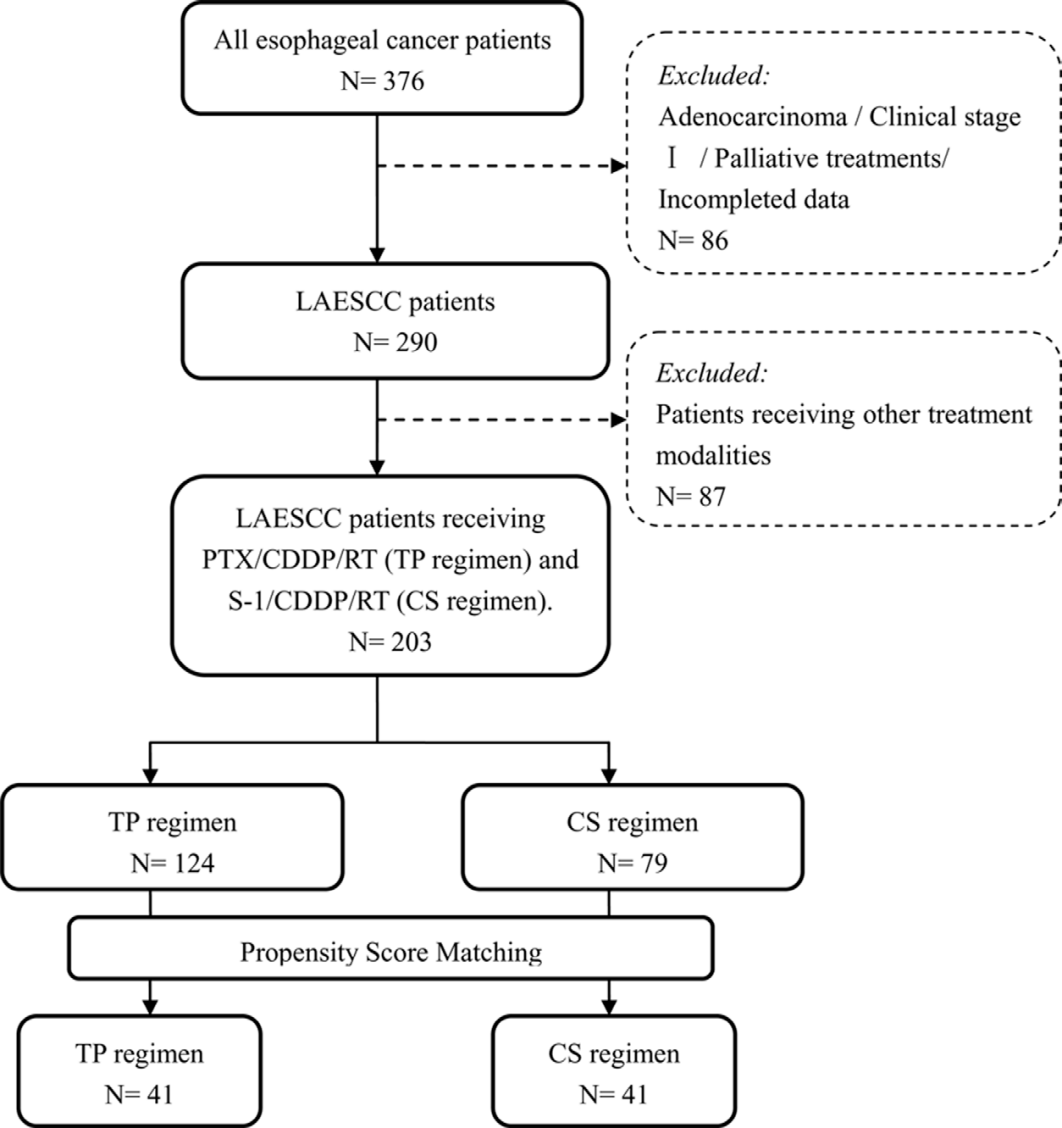
Patient disposition

After PSM analysis, 82 well-balanced unresectable LAESCC patients were available for outcome comparison (41 patients in each group, Figure 1) and the median value of the PSM score for the TP and CS group was 0.423 (range: 0.154–0.840) and 0.422 (range: 0.154–0.814). No significantly demographic difference was observed among these two groups (Table 1). Within the whole series, the median patient age was 58 years (range, 38–77 years). The mean length of the primary tumor was 6.2 cm (range, 2.0–12.5 cm). Nearly half of the tumors were located in the upper-third of the esophagus (46.3%). Fifty-eight (70.7%) patients had stage II-III diseases while 24 (29.3%) patients were diagnosed with stage IVa. Reasons for indication of definitive CCRT for stage II ESCC patients were: cervical esophagus (*n* = 21) and rejection of esophagectomy (*n* = 4).

**Table 1:** Patients’ background characteristics

Characteristic	Total (*n* = 82), %	*PTX/CDDP/RT (n= 41), %*	*S-1/CDDP/RT (n= 41),%*	*P* value
*Age(years)*				0.823
age < 58	35 (42.7)	18 (43.9)	17 (41.5)	
age ≥ 58	47 (57.3)	23 (56.1)	24 (58.5)	
*Gender*				0.532
Female	12 (14.6)	5 (12.2)	7 (17.1)	
Male	70 (85.4)	36 (87.8)	34 (82.9)	
*ECOG Performance Status*				1.000
0–1	58 (70.7)	29 (70.7)	29 (70.7)	
2	24 (29.3)	12 (29.3)	12 (29.3)	
*T Stage*				0.659
T_3_	40 (48.8)	21 (51.2)	19 (46.3)	
T_4_	42 (51.2)	20 (48.8)	22 (53.7)	
*N Stage*				0.656
N_0_	46 (56.1)	24 (58.5)	22 (53.7)	
N_1_	36 (43.9)	17 (41.5))	19 (46.3)	
*M Stage*				0.627
M_0_	58 (70.7)	30 (73.2)	28 (68.3)	
M_1a_	24 (29.3)	11 (26.8)	13 (31.7)	
*Clinical Stage (AJCC 2002)*				0.787
Stage II	25 (30.5)	12 (29.3)	13 (31.7)	
Stage III	33 (40.2)	18 (43.9)	15 (36.6)	
Stage IV_a_	24 (29.3)	11 (26.8)	13 (31.7)	
*Tumor Location*				0.811
Upper-third	38 (46.3)	19 (46.3)	19 (46.3)	
Middle-third	30 (36.6)	16 (39.0)	14 (34.1)	
Lower-third	14 (17.1)	6 (14.7)	8 (19.6)	
*Histological Differentiation*				0.890
Well differentiated	19 (23.2)	10 (24.4)	9 (22.0)	
Fairly differentiated	37 (45.1)	19 (46.3)	18 (43.9)	
Poorly differentiated	26 (31.7)	12 (29.3)	14 (34.1)	
*Tumor Length (cm)*				1.000
< 5	32 (39.0)	16 (39.0)	16 (39.0)	
≥ 5	50 (61.0)	25 (61.0)	25 (61.0)	
*Weight Loss in 6 months*				0.810
≤ 10%	57 (69.5)	29 (70.7)	28 (68.3)	
> 10%	25 (30.5)	12 (29.3)	13 (31.7)	

Abbreviations: n: number of patients; PTX: Paclitaxel: CDDP: Cisplatin; RT: radiotherapy; ECOG: Eastern Cooperative Oncology Group. Upper: including cervical and upper thoracic portion; Middle: Mid-thoracic portion; Lower: including lower thoracic and distal esophagus.

### Treatment compliance and tumor response

All patients in the TP group completed the first cycle of chemotherapy. Three patients refused the second cycle of chemotherapy for occurring grade 4 leukocytopenia, and these patients also refused radiotherapy. Five (12.2%) patients required a dose reduction in the second cycle of chemotherapy for hematological toxicities. Thirty-eight patients completed radiotherapy, including 3 patients with radiation delay and 1 patient with fatal esophagitis. Thus, 29 (70.7%) patients completed the preplanned CCRT on schedule.

Among the 41 patients in the CS group, 39 (95.1%) patients received the full dose of radiotherapy, whereas two patients required radiation delay or dose reduction due to grade 3 or higher esophagitis. Two additional patients required a dose reduction of S-1 for hematological toxicities. Thus, a total of 37 (90.2%) patients completed S-1/CDDP/RT without changing the treatment dose. The difference in treatment compliance during CCRT between the two groups was statistically significant (*P* = 0.026). In addition, lengths of hospital stay and hospitalization expenses during CCRT course were also collected for the two groups. The median length of hospital stay and medical cost for TP group were 49 days (range: 44–61 days) and 12.70 thousand dollars person-times (range: 10.50-17.20 thousand dollars). The corresponding figures for CS group were 48 days (range: 44–57 days) and 12.90 thousand dollars person-times (range: 10.80–16.10 thousand dollars). By comparing to the TP group, CS group significantly decreased the length of hospital stay (*P* = 0.025) and the inpatient expenses was not statistically significant (*P* = 0.904). After completing CCRT, 11 (26.8%) patients in the TP group completed the additional full 4 cycles of chemotherapy while 18 (43.9%) patients in the CS group received the additional 4 cycles of maintenance chemotherapy. This difference was not statistically significant (*P* = 0.106). The median number of chemotherapy cycles was five for both treatment regimens while the total number of cycles delivered was 186 and 199 for TP and CS groups, respectively.

The tumor response was documented using RECIST. In the TP group, complete response (CR) was observed in 21 patients (51.2%), partial response (PR) in 13 patients (31.7%), stable disease (SD) in 4 patients (9.8%), and progressive disease (PD) in 3 (7.3%) patients, yielding an objective response rate (ORR) of 82.9%. In addition, in the CS group, 34 patients achieved an ORR of 82.9% (CR = 20 and PR = 14) and 2 patients exhibited PD. No significant difference in the CR and ORR rates were observed between the two groups (*P* = 0.825 and 1.000, respectively).

### Acute and late toxic reactions

Acute treatment-related toxicity could be evaluated in all cases (Table 2). In general, patients in the TP group suffered from more treatment toxicities than those who received the CS regimen. There was grade 3–4 hematologic toxicity in 48.8% of patients receiving TP versus 31.7% of patients who received CS (*P* = 0.115). TP was associated with a significantly higher rate of severe and fatal neutropenia than CS (*P* = 0.011). The occurrence of severely non-hematologic toxicity was not significantly different between the two groups. A comparison of the total incidence of hematological toxicities revealed that the CS group also had a significant lower incidence of neutropenia over the TP group (*P* = 0.046). For non-hematological toxicity, the TP group demonstrated a significantly higher incidence of neurotoxicity compared with the CS group (*P* = 0.034). During maintenance chemotherapy, the TP regimen had a higher incidence of nausea/vomiting compared with the CS group (*P* = 0.046). Hematological toxicities were comparable in both treatment groups. There were no treatment-related toxic deaths during the treatment course.

**Table 2:** Treatment-related toxicities

Event	PTX/CDDP/RT (*n* = 41), %		S-1/CDDP/RT (*n* = 41), %		*P* value	
*Events of grade ≥ 3 during CCRT*						
Leucocytopenia	14 (34.1)		10 (24.4)		0.332	
Neutropenia	20 (48.8)		9 (22.0)		*0.011*	
Anemia	6 (14.6)		3 (7.3)		0.480	
Thrombocytopaenia	5 (12.2)		4 (9.8)		1.000	
Esophagitis	4 (9.8)		2 (4.9)		0.672	
Anorexia	1 (2.4)		2 (4.9)		1.000	
Nausea/Vomiting	3 (7.3)		2 (4.9)		1.000	
Diarrhea	2 (4.9)		1 (2.4)		1.000	
Fatigue	3 (7.3)		2 (4.9)		1.000	
Pneumonitis	1 (2.4)		2 (4.9)		1.000	
*Events of any grade during CCRT*						
Leucocytopenia	35 (85.4)		28 (68.3)		0.067	
Neutropenia	37 (90.2)		30 (73.2)		*0.046*	
Anemia	25 (61.0)		27 (65.9)		0.647	
Thrombocytopaenia	16 (39.0)		14 (34.1)		0.647	
Esophagitis	34 (82.9)		38 (92.7)		0.177	
Anorexia	22 (53.7)		28 (68.3)		0.174	
Nausea/Vomiting	23 (56.1)		17 (41.5)		0.185	
Diarrhea	17 (41.5)		12 (29.3)		0.248	
Fatigue	26 (63.4)		22 (53.7)		0.370	
Pneumonitis	7 (17.1)		7 (17.1)		1.000	
Liver function	5 (12.2)		8 (19.5)		0.364	
Nephrotoxicity	2 (4.9)		1 (2.4)		1.000	
Neurotoxicity	8 (19.5)		1 (2.4)		*0.034*	
Constipation	2 (4.9)		4 (9.8)		0.672	
Mucositis	3 (7.3)		2 (4.9)		1.000	

Events of any grade during maintenance CT		PTX/CDDP/RT (*n*= 38), %		S-1/CDDP/RT (*n*= 39), %		*P* value
Leucocytopenia		15 (39.5)		9 (23.1)		0.120
Neutropenia		11 (28.9)		14 (35.9)		0.515
Anemia		4 (10.5)		10 (25.6)		0.086
Thrombocytopaenia		5 (13.2)		8 (20.5)		0.389
Nausea/Vomiting		15 (39.5)		7 (17.9)		*0.037*
Diarrhea		6 (15.8)		4 (10.3)		0.702
Fatigue		7 (18.4)		5 (12.8)		0.498
Liver function		3 (7.9)		2 (5.1)		0.976
Nephrotoxicity		5 (13.2)		3 (7.7)		0.680
*Late grade ≥ 3 toxicity*						
Esophagus		4 (9.8)		5 (12.2)		1.000
Lung		2 (4.9)		1 (2.4)		1.000
Heart		1 (2.4)		2 (4.9)		1.000

Late grade ≥ 3 esophageal toxicities were observed in four patients in the TP group and 5 patients in the CS group. The incidences of late esophageal, lung and heart toxicities showed no significant difference between the two groups.

### Survival and prognostic analysis

The median follow-up period was 28.4 months. The median OS of the overall population was 21.6 months (95% CI: 15.4–27.8 months), and the 1-year OS rates were 63.4% and 62.8% for TP group and CS group, respectively. The 3-year OS rates were 32.4% and 32.1% for TP and CS group, respectively. The difference in OS was not statistically significant (*P* = 0.796).

**Figure 2:**
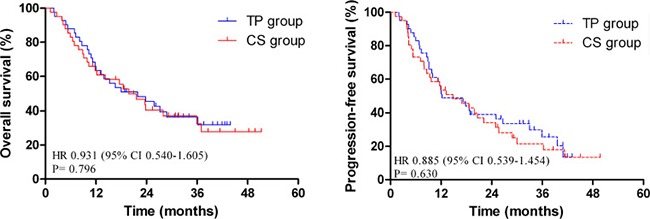
Comparison of overall survival (OS) and progression-free survival (PFS) in matched patients

During the follow-up period, 31 patients had progressive disease in the TP group. Primary failure sites included the following: 14 locoregional and residual disease, 12 distant, and 5 in both sites. In the CS group, 32 patients experienced treatment failure, and the corresponding figures were 17 (41.5%), 10 (24.4%) and 5 cases (12.2%). The median PFS was 13.2 months (95% CI: 7.4–19.1 months), and the 1- and 3-year PFS rates were 51.2%, 24.9% and 53.6%, 18.9% for the TP group and CS group, respectively. No statistically significant difference was found when comparing group survival times for PFS (*P* = 0.630, Figure 2).

Univariate analyses were performed to assess the predictive capability of each variable (Table 3). These results suggested that several covariates were significantly associated with OS: ECOG PS (HR 2.416, *P* = 0.002), T stage (HR 4.101, *P* = 0.000), N stage (HR 2.387, *P* = 0.002), M stage (HR 2.129, *P* = 0.010), clinical stage (HR 1.892, *P* = 0.000), and clinical response (HR 3.898, *P* = 0.000). The variables significantly associated with PFS were: ECOG PS (HR 1.976, *P* = 0.013), T stage (HR 2.570, *P* = 0.000), N stage (HR 2.304, *P* = 0.002), clinical stage (HR 1.537, *P* = 0.009), and clinical response (HR 3.057, *P* = 0.000).

**Table 3:** Univariate analysis demonstrating factors associated with OS and PFS in matched patients

Factor	Cases (n)	OS *p*-value	HR (95% CI)	PFS *p*-value	HR (95% CI)
*Treatment regimen*		0.796	1.074 (0.624–1.851)	0.630	1.129 (0.688–1.853)
TP	41				
CS	41				
*Age*		0.168	0.674 (0.385–1.181)	0.116	0.664 (0.399–1.106)
age < 58	35				
age ≥58	47				
*Sex*		0.428	1.338 (0.652–2.748)	0.498	1.265 (0.641–2.499)
Female	12				
Male	70				
*ECOG PS*		0.002	2.416 (1.366–4.276)	0.013	1.976 (1.156–3.379)
0–1	58				
2	24				
*T Stage*		0.000	4.101 (2.269–7.413)	0.000	2.570 (1.546–4.273)
T3	40				
T4	42				
*N Stage*		0.002	2.387 (1.365–4.172)	0.002	2.304 (1.369–3.876)
N0	46				
N1	36				
*M Stage*		0.010	2.129 (1.195–3.792)	0.058	1.711 (0.982–2.981)
M0	58				
M1	24				
*Clinical Stage*		0.000	1.892 (1.321–2.708)	0.009	1.537 (1.111–2.125)
II	25				
III	33				
IV_a_	24				
*Tumor Location*		0.173	1.305 (0.890–1.913)	0.082	1.364 (0.061–1.937)
Upper-third	38				
Middle-third	30				
Lower-third	14				
*Differentiation*		0.255	0.806 (0.556–1.169)	0.327	0.842 (0.597–1.187)
Well	19				
Fairly	37				
Poorly	26				
*Tumor Length (cm)*		0.252	1.396 (0.789–2.470)	0.082	1611 (0.941–2.758)
< 5	32				
≥ 5	50				
*Weight Loss*		0.479	1.233 (0.690–2.203)	0.683	1.119 (0.653–1.919)
≤ 10%	57				
> 10%	25				
*Clinical Response*		0.000	3.898 (2.171–6.997)	0.000	3.057 (1.825–5.121)
CR	41				
Non-CR	41				

Abbreviations: n: number of patients; OS: overall survival; PFS: progression-free survival; HR: hazard ratio; CI: confidence interval, CR: complete response.

To identify independent prognostic factors, the factors that were found to be significant using univariate analysis were subjected to multivariate analysis. Multivariate analysis revealed that T stage (*P* = 0.009 and 0.042, respectively), N stage (*P* = 0.027 and 0.014, respectively) and clinical response (*P* = 0.002 and 0.012, respectively) were independent factors affecting OS and PFS (Table 4).

**Table 4:** Multivariate analysis of prognostic factors for matched patients

Endpoint	Factor	*p*-value	HR (95% CI)
***OS***	T Stage	0.009	3.420 (1.365–8.569)
	N Stage	0.027	2.270 (1.098–4.692)
	M Stage	0.072	3.571 (0.891–14.307)
	Clinical Stage	0.229	0.498 (0.160–1.551)
	ECOG PS	0.128	1.596 (0.875–2.912)
	Clinical Response	0.002	2.860 (1.488–5.497)
***PFS***	ECOG PS	0.239	1.413 (0.795–2.511)
	T Stage	0.042	2.083 (1.026–4.229)
	N Stage	0.014	2.295 (1.184–4.450)
	Clinical Stage	0.718	0.904 (0.522–1.566)
	Clinical Response	0.012	2.061 (1.175–3.613)

Abbreviations: OS: overall survival; PFS: progression-free survival; HR: hazard ratio; CI: confidence interval.

Furthermore, the 1- and 3-year OS rates and the median OS time for 124 patients with TP regimen were 76.9%, 36.1%, and 24.9 months, respectively, and 71.6%, 30.6%, 23.1 months, respectively, for all patients received CS regimen. The 1- and 3-year PFS rates were 71.4%, 26.3% and 66.8%, 23.8% for the TP group and CS group, respectively. No significant difference was found for both OS and PFS in two groups (*P* = 0.660 and 0.684, respectively). Multivariate analysis among all patients revealed that T stage (OS: HR 1.846, *P* =0.002; PFS: HR 1.758, *P* =0.002) and clinical response (OS: HR 2.783, *P* = 0.000; PFS: HR 2.207, *P* = 0.000) were strong prognostic factors associated with OS and PFS. In addition, M stage (HR 2.482, *P* = 0.016) and weight loss (HR 1.423, *P* = 0.038) were significant factors associated with PFS (Supplementary Tables 2 and 3).

## DISCUSSION

It has been shown previously that TP regimen combined with RT yielded favorable response rates and comparable survival outcomes [7, 8]. However, treatment-related toxicities need to be taken into consideration while making the treatment plan. S-1 was an orally active fluoropyrimidine with enhanced anticancer effects and reduced gastrointestinal toxicities. It has been widely used in a variety of solid tumors, including esophageal cancer [17, 18]. In a phase II trial performed by Cho et al., 30 locally advanced or metastatic ESCC patients were assigned to receive S-1 and CDDP at doses of 70 mg/m^2^/day for 14 days and 70 mg/m^2^ on day 1, respectively, every 3 weeks. After CCRT, additional chemotherapy was administered up to six cycles [19]. The ORR was observed in 74.1% (20/27) patients. In patients at stages II–III, the median PFS and OS were 10.6 and 23.0 months, respectively, while for patients with metastatic diseases (4 patients were diagnosed with stage IVb), the median PFS and OS were 5.4 and 11.6 months, respectively. The main hematological toxicity was neutropenia (19%, 10/54) and the major non-hematological toxicities were asthenia and vomiting, which were mostly observed for grades 1 and 2. In another prospective study, 116 LAESCC patients were allocated to receive a 30Gy RT over 3 weeks plus daily oral S-1 (80 mg/m^2^/day) for 2 weeks and a 24-hour CDDP infusion (70 mg/m^2^) on day 8, and an identical course administered after a 2-week break [14]. 91.4% (106/116) of patients completed the CCRT course. ORR was achieved in 89.7% patients. The 1- and 5-year OS rates were 78.2% and 29.8%, respectively, and the median PFS was 1.2 years. Grade 3 and 4 neutropenia was observed in 28.4% and 9.5% of patients, respectively. Non-hematologic toxicity was moderate. Our data were consistent with these studies. However, the incidence of total neutropenia in the TP group was not significantly higher during maintenance chemotherapy in our study, one probable reason to explain this phenomenon was that chemotherapy combined with radiation therapy had superposition effect during CCRT, which would increase the incidence of toxic reactions [19].

To date, although there were no direct comparisons between TP and CS for esophageal cancer, a similar comparison of PTX versus S-1 was observed in non-small cell lung cancer (NSCLC) patients. A multicenter phase 3 study randomizing stage IIIb-IV NSCLC patients to PTX/carboplatin versus S-1/carboplatin chemotherapy was performed [21]. The median OS was 15.2 versus 13.3 months in the S-1/carboplatin and PTX/carboplatin arm, with 1-year OS rates of 57.3% and 55.5%, respectively. The rates of grade 3/4 leukopenia or neutropenia, febrile neutropenia, alopecia, and neuropathy were more frequently observed in the PTX/carboplatin group. It was concluded that S-1 with carboplatin was not inferior in terms of OS compared with carboplatin and PTX in patients with advanced NSCLC and was thus a valid treatment option.

In the present study, we confirmed that the T stage was a strong prognostic factor for both OS and PFS before and after PSM analysis, which was consistent with previous reports in the literature [22, 23]. In addition, patients who were evaluated as a complete response were also confirmed to have longer survival times compared with non-CR patients [24].

This study has some limitations. One limitation of the current study is the retrospective nature of the study design. Although propensity score matching was performed to reduce potential influence, some unmeasured factors might have effects on the final results. Furthermore, another limitation of current study was the relatively small sample size and low-level of power performed in a single center.

In conclusion, both the TP and CS regimens yielded encouraging survival outcomes and manageable side effects. However, the CS regimen could significantly improve treatment compliance, reduce hematological toxicities and lengths of hospital stay. On the basis of the findings of this propensity score matching analysis, future prospective trials in large cohorts are highly needed to confirm the findings (i.e., NCT01704690).

## MATERIALS AND METHODS

### Patient work-up

This retrospective study was approved by the ethical committee of Zhejiang Provincial People's Hospital and the written informed consent was obtained from all patients. Between January 2009 and December 2013, 376 consecutive patients with newly diagnosed esophageal cancer were reviewed at our cancer center. Inclusion in our review included the following criteria: i) a histological diagnosis of esophageal squamous cell carcinoma; ii) disease stages of II to IVa according to the 2002 (version 6.0) American Joint Committee on Cancer staging system; iii). unresectable diseases or refusal of surgery after being discussed by the multidisciplinary treatment team; iv) an ECOG PS of at least 2; v) no evidence of severe organ dysfunction; and vi) no prior chest radiation or chemotherapy received.

### Treatment

#### Radiotherapy

Radiotherapy was delivered with 6–10 Mv X-ray accelerators using the three-dimensional conformal technique (3D-CRT). The gross tumor volume (GTV) included the primary tumor and any enlarged lymph nodes. For regional lymph nodes, the supraclavicular, upper mediastinal, and subcarinal lymph nodes were irradiated for tumors in the proximal esophagus. The mediastinal and perigastric lymph nodes were included for tumors of the middle or lower esophagus, in which the celiac lymph nodes were added for lower-segment cancers. The clinical target volume (CTV) was defined as the GTV plus a 4–5 cm margin in the superior and inferior directions, 1 cm in the left and right directions and 0.8–1 cm in the anterior and posterior directions. The planning target volume (PTV) was defined as the CTV plus a 5–10 mm margin around the CTV. The preplanned total dose was 60.0 Gy, which was administered in 30 fractions of 2.0 Gy once-daily fractions for 5 days per week.

### Chemotherapy

Two cycles of concurrent chemotherapy were delivered with RT. CDDP (30 mg/m^2^) was administered intravenously on days 1–3 and days 29–31 for all enrolled patients [25]. The chemotherapy regimen of TP group patients was as follows: PTX 135 mg/m^2^, *i.v*., was administered for 3 hours on day 1 and day 29 with standard premedications. The chemotherapy regimen of CS group patients: S-1 was administered orally twice daily for 2 weeks at a dose of 70 mg/m^2^/day. Patients who showed a response greater than that of stable disease underwent additional four cycles of chemotherapy.

### Dose modification

Dose modifications were considered on a weekly basis during CCRT. Chemotherapy was delayed for acute toxicities until recovery to grades ≤ 2 and/or the dose was reduced for grade 3 or higher hematological toxicity. PTX was reduced to 80% in the second course if any of the following phenomena occurred: grade 3 leukocytopenia with fever or grade 4 leukocytopenia. The granulocyte colony-stimulating factor (G-CSF) was used to treat the occurrence of febrile neutropenia. If the creatinine clearance decreased to less than 50 mL/min, then the dose of CDDP was reduced to 75%. S-1 was suspended due to excessive toxicity or patient request. In cases of severe toxicity related with S-1, interruptions were allowed up to 14 days. If the side effects did not return to grades ≤ 2, then a dose reduction by 60 mg/m^2^/day was allowed, but no more than one dose reduction was permitted. Irradiation was interrupted for grade ≥ 3 esophagitis, grade 3 leukocytopenia with fever, or grade 4 leukocytopenia. Radiotherapy was restarted when toxicities recovered to grades ≤ 2.

### Treatment assessment and follow-up

Treatment related toxicity was evaluated according to the common toxicity criteria for adverse events version 3.0 (CTCAE v3.0). We evaluated the clinical response of patients using the Response Evaluation Criteria in Solid Tumors (RECIST) 3–4 weeks after the completion of CCRT. Follow-up was regularly performed at 3 months during the first year, then every 6 months for 2 additional years, and then on a yearly basis. Treatment failure was defined as any sign of recurrent disease, which could be locoregional, distant, or both. We assessed failure models on post-treatment esophagogram, endoscopy, CT, or PET/CT (if available) scans and compared these data with the original CT-based treatment plans.

### Statistical analysis

To reduce bias of potential confounders between the two groups, propensity score matching was performed to generate two treatment cohorts with a balanced distribution of baseline characteristics. The following variables were included in a logistic regression model, regardless of their individual statistical significance: gender; ECOG PS; T stage; M stage; clinical stage; tumor length; and weight loss over the nearly 6 month-study period. The potential predictors that were not statistically significant were removed (Supplementary Table 1), and propensity scores were calculated from the logistic regression refit to the reduced variable group. Only patients matched with propensity scores were included in the subsequent analyses.

Covariate balances between the two sets were examined by *t* test (continuous variable), Mann-Whitney u test (abnormal distribution variable), χ^2^ test or Fisher's exact test (categorical variable) as appropriate. OS was determined as the time between the first day of treatment and the last follow-up or the date of death. PFS was defined as the internal between the date of treatment initiation and the date of documented failure or the date of the last follow-up for those remaining. Survival curves were determined using the Kaplan-Meier method. Predictive factors of survival were analyzed using univariate analysis and further evaluated in the multivariate Cox regression model to estimate the hazard ratio (HR) with a 95% confidence interval (CI). All statistical calculations were performed using STATA 12.0 (Stata Corporation, College Station, TX, USA) and Statistical Package for the Social Sciences (SPSS 20.0, Inc., Chicago; for Windows). A *P* value < 0.05 was considered statistically significant.

## SUPPLEMENTARY MATERIALS TABLES


