# Supplementary material for: Comparative study of cisplatin-based definitive concurrent chemoradiotherapy with S-1 versus paclitaxel for unresectable locally advanced esophageal squamous cell carcinoma
Source: Oncotarget. 2017 Mar 14;8(23):37080–90. doi: 10.18632/oncotarget.16180 (PMC5514892; doi:10.18632/oncotarget.16180)
Supplement: Supplementary file 1 [file oncotarget-08-37080-s001.pdf]

Comparative study of cisplatin-based definitive concurrent chemoradiotherapy with S-1 versus paclitaxel for unresectable locally advanced esophageal squamous cell carcinoma

Supplementary Materials

Supplementary Table 1: Patients' baseline characteristics before propensity score matching

Characteristic	*PTX/CDDP/RT (n = 124), %*	*S-1/CDDP/RT (n = 79), %*	*P* value
Age(years)			0.312
age < 57	56 (45.2)	30 (38.0)	
age ≥ 57	68 (54.8)	49 (62.0)	
Gender			0.025
Female	21 (16.9)	24 (30.4)	
Male	103 (83.1)	55 (69.6)	
ECOG Performance Status			0.023
0–1	98 (79.0)	51 (64.6)	
2	26 (21.0)	28 (35.4)	
T Stage			0.010
T_3	67 (54.0)	28 (35.4)	
T_4	57 (46.0)	51 (64.6)	
N Stage			0.826
N_0	49 (39.5)	30 (38.0)	
N_1	75 (60.5)	49 (62.0)	
M Stage			0.025
M_0	94 (75.8)	48 (60.8)	
$M1_a$	30 (24.2)	31 (39.2)	
Clinical Stage (AJCC 2002)			0.012
Stage II	12 (9.7)	19 (24.1)	
Stage III	82 (66.1)	39 (49.4)	
Stage IV_a	30 (24.2)	21 (26.5)	
Tumor Location			0.780
Upper-third	45 (36.3)	25 (31.6)	
Middle-third	46 (37.1)	34 (43.0)	
Lower-third	26 (21.0)	17 (21.5)	
Multi-section	7 (5.6)	3 (3.9)	
Histological Differentiation			0.186
Well differentiated	21 (16.9)	13 (16.5)	
Fairly differentiated	46 (37.1)	39 (49.4)	
Poorly differentiated	57 (46.0)	27 (34.1)	
Tumor Length (cm)			0.030
< 5	28 (22.6)	31 (39.2)	
≥ 5	86 (77.4)	48 (60.8)	
Weight Loss in 6 months			0.026
≤ 10%	79 (63.7)	62 (78.5)	
> 10%	45 (36.3)	17 (21.5)	

n: number of patients; PTX: Paclitaxel: CDDP: Cisplatin; RT: radiotherapy; ECOG: Eastern Cooperative Oncology Group. Upper: including cervical and upper thoracic portion; Middle: Mid-thoracic portion; Lower: including lower thoracic and distal esophagus.

Supplementary Table 2: Univariate analysis demonstrating factors associated with OS and PFS for all patients

Factor	Cases (*n*)	OS *p*–value	HR (95% CI)	PFS *p*–value	HR (95% CI)
Treatment regimen		0.661	1.079 (0.767–1.519)	0.687	1.067 (0.778–1.463)
TP	124				
CS	79				
Age		0.601	1.094 (0.780–1.535)	0.569	1.095 (0.801–1.196)
age < 57	86				
age ≥ 57	117				
Sex		0.195	0.754 (0.492–1.156)	0.577	0.900 (0.621–1.304)
Female	45				
Male	158				
ECOG PS		0.066	1.413 (0.978–2.043)	0.026	1.473 (1.048–2.071)
0–1	149				
2	54				
T Stage		0.000	2.251 (1.594–3.179)	0.000	2.166 (1.578–2.973)
T3	95				
T4	108				
N Stage		0.698	1.070 (0.759–1.508)	0.519	0.901 (0.658–1.236)
N0	81				
N1	122				
M Stage		0.000	1.917 (1.351–2.721)	0.000	2.142 (1.547–2.965)
M0	141				
M1	62				
Clinical Stage		0.006	1.522 (1.127–2.055)	0.001	1.652 (1.243–2.195)
II	30				
III	111				
IV_a	62				
Tumor Location		0.448	1.080 (0.886–1.9317)	0.286	1.107 (0.918–1.335)
Upper-third	70				
Middle-third	80				
Lower-third	43				
Multi-section	10				
Differentiation		0.628	0.946 (0.754–1.186)	0.650	0.953 (0.772–1.175)
Well	34				
Fairly	85				
Poorly	84				
Tumor Length (cm)		0.836	1.039 (0.721–1.499)	0.782	1.049 (0.747–1.474)
< 5	32				
≥ 5	50				
Weight Loss		0.042	1.466 (1.013–2.065)	0.004	1.619 (1.162–2.255)
≤ 10%	59				
> 10%	134				
Clinical Response		0.000	3.119 (2.199–4.424)	0.000	2.556 (1.865–3.502)
CR	141				
Non-CR	62				

n: number of patients; OS: overall survival; PFS: progression–free survival; HR: hazard ratio; CI: confidence interval, CR: complete response.

Supplementary Table 3: Multivariate analysis of prognostic factors for all patients

Endpoint	Factor	*p*-value	HR (95% CI)
OS	T Stage	0.002	1.846 (1.241–2.745)
	M Stage	0.091	1.973 (0.898–4.335)
	Clinical Stage	0.593	0.840 (0.444–1.592)
	Weight Loss	0.148	1.301 (0.911–1.858)
	Clinical Response	0.000	2.783 (1.932–4.007)
PFS	ECOG PS	0.458	1.142 (0.804–1.623)
	T Stage	0.002	1.758 (1.222–2.529)
	M Stage	0.016	2.482 (1.187–5.188)
	Clinical Stage	0.363	0.760 (0.420–1.373)
	Weight Loss	0.038	1.423 (1.020–1.984)
	Clinical Response	0.000	2.207 (1.584–3.074)

OS: overall survival; PFS: progression-free survival; HR: hazard ratio; CI: confidence interval.
